# Children and young people's experiences of living with developmental coordination disorder/dyspraxia: study protocol for a qualitative evidence synthesis

**DOI:** 10.12688/hrbopenres.12958.4

**Published:** 2021-01-18

**Authors:** Áine O'Dea, Susan Coote, Katie Robinson

**Affiliations:** 1 School of Allied Health, University of Limerick, Castletroy, Limerick, Ireland; 2 Health Research Institute, University of Limerick, Castletroy, Limerick, Ireland; 3 Ageing Research Centre, University of Limerick, Castletroy, Limerick, Ireland

**Keywords:** Developmental Coordination Disorder, children, young people, meta-ethnography

## Abstract

**Background**

Children with developmental coordination disorder (DCD) face significant challenges to deal with everyday activities due to underlying motor proficiency difficulties. These challenges affect children and young people’s participation; that is, involvement in daily life situations. Recent years have seen a growing body of qualitative research examining children’s experiences of living with DCD.  Meta-ethnographic synthesis offers a rigorous approach to bring together the findings of discrete qualitative studies to be synthesised in order to advance the conceptual understanding of living with DCD, which is not well conceptualised in the literature to date. Conducting a meta-ethnographic synthesis will help to illuminate the meaning of children and young people’s experiences of DCD regarding their involvement in everyday activities and situations.

**Aim **

This study aims to systematically review and synthesise qualitative literature regarding children and young people’s experiences and views of everyday life and living with DCD.

**Methods**

The method of qualitative evidence synthesis that will be followed in this review is a meta-ethnography. The eMERGe and PRISMA reporting guidelines will be adhered to. Ten databases will be searched; Academic Search Complete, AMED, CINAHL, ERIC, MEDLINE, PsychArticles, PsychInfo, EMBASE, SPORTDiscus, and Web of Science. The Joanna Briggs Institute Checklist will be used by two independent reviewers to appraise all included papers. PROSPERO registration number CRD42019129178

**Discussion**

The findings of this meta-ethnography will endeavour to inform future research, policy and practice. In particular, the results will help to inform the design of future complex interventions to meet the needs of children and young people with DCD. Dissemination will involve the publication of the results in a peer-reviewed journal. Increasingly researchers and policymakers are calling for services to be informed by the perspective and voice of children with DCD. Therefore, a policy brief will be published so that the findings are widely available.

## Introduction

Children with developmental coordination disorder (DCD) struggle to master numerous everyday activities that involve motor coordination ( APA, 2013), for example, self-care, leisure and academic activities including feeding, sports, and writing ( Summers *et al*., 2008; Van der Linde *et al*., 2015). The core features of this diagnostic condition are; A) learning and execution of coordinated motor skills is below the expected level for age given the opportunity for skill learning; B) motor skill difficulties significantly interfere with activities of daily living and impact academic/school, leisure and play; C) onset is in the early developmental period; and D) motor skill difficulties are not better explained by intellectual delay, visual impairment or other neurological conditions that affect movement ( APA, 2013). Prevalence rates of DCD are considered to be between 5 and 6% of the population ( Blank *et al*., 2019). However, international prevalence rates vary from between 1.8% to 20% of the paediatric population ( Valentini *et al*., 2015). The reasons from such variance are the prevalence rate is associated with the diversity of methods used, such as sample population, measurement tools, and cut-off percentiles for DCD ( Valentini *et al*., 2015).

The consequences of DCD are enduring ( Blank *et al*., 2019), and affect children and young people’s participation; that is, involvement in daily life situations ( WHO, 2007) across, social, academic, work, vocational and leisure areas ( O'Dea & Connell, 2016; Kirby *et al*., 2011). Adverse, secondary health outcomes associated with DCD include poor cardiovascular health and obesity ( Cairney *et al*., 2010), and mental health difficulties such as anxiety and depression ( Harrowell *et al*., 2017; Pratt & Hill, 2011). Secondary health outcomes persist across the lifespan with adults with DCD describing higher levels of depression and anxiety ( Hill & Brown, 2013).

Evidence regarding the effectiveness of interventions to treat DCD is not clear due to the quality of the available evidence ( Miyahara *et al*., 2020). A recent meta-review of systematic reviews and meta-analyses of interventions for children with DCD found that of the eight included reviews the methodology of only one ( Miyahara *et al*., 2017), was judged using AMSTAR 2 terminology to be acceptably high ( Miyahara *et al*., 2020). This meta-review also highlighted that the conclusions of these recent reviews are not consistent. This inconsistency creates challenges for practitioners. The highest quality review included in the meta-review was a Cochrane review that found no strong evidence to support the efficacy of task-oriented interventions for children and young people with DCD ( Miyahara *et al*., 2017), an approach commonly used in practice ( Withers *et al.* 2017). In contrast, Smits-Engelsman *et al.* (2018), systematic review and meta-analysis found that activity-oriented and body function oriented interventions can have a positive effect on motor function and skills. Of note, the authors suggest that the results should be interpreted with caution, given the variance in methodology quality and the large confidence intervals ( Smits-Engelsman *et al*., 2018). Furthermore, the research evidence regarding which interventions are effective at improving outcomes addressing participation in everyday life situations for children and young people with DCD is not clear ( O’Dea *et al*., 2019). Given the current state of the evidence base, the robust development and evaluation of interventions for children with DCD have been identified as a priority ( Miyahara *et al*., 2017; O’Dea *et al*., 2019).

The International Classification of Functioning (ICF) framework provides a contemporary vision of health and functioning ( World Health Organisation, 2001). It proposes that disability results from the interaction between environmental and personal factors ( World Health Organisation, 2001). Research in DCD has adopted the terminology of the ICF and considers outcomes at the activity and participation level important ( Smits-Engelsman *et al*., 2018). Reflecting these perspectives, international clinical practice guidelines for DCD state that intervention should consider the child and family identified goals, related to activity and participation within the environmental context ( Blank *et al*., 2019). Therefore, the perspectives of children and young people with DCD should be central to intervention development in practice and research. Qualitative research has much to offer clinicians and researchers working with children and young people with DCD. It can illuminate the meaning of their experiences regarding their involvement in everyday activities and situations i.e. their participation ( Taylor & Francis, 2013), and their perspectives, views and experiences of their life situations ( Söderbäck *et al*., 2011).

In the past, there has been greater attention to researching the perceptions and experiences of parents of children with DCD rather than focusing on the experience of the children or young people themselves. This body of research addresses topics such as raising a child with DCD and accessing services and support for their child with DCD ( Maciver *et al*., 2011; Missiuna *et al*., 2006; Missiuna *et al*., 2007; Morgan & Long, 2012; Novak *et al*., 2012). Importantly, findings from qualitative studies suggest that parental perceptions are different to those of children and young people with DCD ( Jasmin *et al*., 2018; Morgan & Long, 2012; Timler *et al*., 2018), for example, parental assessment in comparison to young people’s self-assessment of motor competence highlights that parents recognise fewer motor difficulties than the young person ( Timler *et al*., 2018). With regard to effective interventions and participation in home and community environments, parents prioritise training and coaching on DCD to help facilitate their child’s learning and autonomy with activities of daily living; whereas, as children with DCD, prioritise aspects such as play ( Jasmin *et al*., 2018; Morgan & Long, 2012). Therefore, qualitative research on the experiences and perspectives of parents of children with DCD should not be considered to represent the experiences and perspectives of children with DCD.

According to the United Nations Convention on the Rights of the Child (1989), children are entitled to express opinions and to have a say in matters that affect their lives ( Children's Rights Alliance, 2010). Similarly, authors ( Lynch & Lynch, 2013; McQuinn *et al*., 2019) and policy documents ( Ombudsman for Children's Office, 2019) state that the perspectives of children and young people with disabilities need to be included in practice and research. Indeed, children and young people value potential contributions with research ( Lynch & Lynch, 2013; Söderbäck *et al*., 2011) and empirical studies indicate that children are capable of contributing their opinions, views and preferences for therapeutic intervention ( Dunford *et al*., 2005). Despite the acknowledgement that children are knowledgeable, capable and proficient research participants ( Christensen & Prout, 2005), children with disabilities continue to be overlooked as active research participants ( Stafford, 2017).

Recent years have seen a growing body of qualitative research examining children’s experiences of living with DCD ( Payne *et al*., 2013) and related topics, such as identity and self-management ( Lingam *et al*., 2014), priorities and preferences for treatment ( Dunford *et al*., 2005), participation ( Jasmin *et al*., 2018) and quality of life ( Zwicker *et al*., 2018). To the best of our knowledge, no qualitative evidence synthesis has integrated children and young people’s subjectively reported experiences of living with DCD. Therefore, a meta-ethnographic approach, informed by Noblit & Hare’s (1988) seven-stage process for conducting a meta-ethnography was chosen as the method of qualitative evidence synthesis for this review. Meta-ethnography offers a methodology for synthesising multiple qualitative research studies. A strength of meta-ethnographic synthesis is the capacity to bring numerous qualitative research studies together to detail a narrative that is greater than the sum of its parts ( Murray & Stanley, 2016). Therefore, a meta-ethnographic synthesis achieves more than aggregation or combination of the included studies. Through translating the findings from the included studies into one another ( Noblit & Hare, 1988), meta-ethnographic synthesis aims to create novel interpretations and conceptual innovation of the phenomena being explored ( Malpass *et al.*, 2009).

Through the process of translating studies into one another ( Noblit & Hare, 1988), the aim of meta-ethnography is to create novel interpretations and conceptual innovation of the phenomenon being studied ( Malpass *et al.*, 2009). Conducting a meta-ethnographic synthesis of existing qualitative studies reporting the experiences of children and young people with DCD will advance our understanding of what it is like to live with DCD, which is not well conceptualised in the literature to date and will identify gaps in the current qualitative literature. Furthermore, qualitative synthesis can help inform the future development and implementation of complex interventions ( France *et al*., 2019a), which is an identified priority for children and young people with DCD ( Camden *et al*., 2019).

## Objective

The principal objective of this study is to systematically review and synthesise qualitative literature regarding children and young people’s experiences and views of everyday life and living with DCD.

## Methods

Qualitative evidence synthesis involves synthesising multiple qualitative primary research studies ( France *et al*., 2019b). Various methods of qualitative evidence synthesis exit, for example, metanarrative, meta-study, critical interpretative synthesis, thematic synthesis and meta-ethnography ( Barnett-Page & Thomas, 2009). A meta-ethnographic approach has been chosen as the method of qualitative evidence synthesis for this review because it is “provides the opportunity for us to carefully consider the relationship between studies, understand the issues and to comprehend the reality of everyday life” ( Noblit & Hare, 1988, p. 77). It requires an interpretive approach to synthesis. The meta-ethnographic synthesis approach of Noblit & Hare (1988) will be employed as described by Cahill *et al.* (2018). It involves a seven-stage process; it moves beyond the collation of qualitative evidence and towards the generation of new understandings. In health services research, meta-ethnographic synthesis has become a popular methodology for qualitative evidence synthesis ( Ring *et al*., 2011), and it is the most popular approach to qualitative evidence in healthcare ( Cahill *et al*., 2018). However, the methodology must be conducted and reported proficiently ( Cahill *et al*., 2018), if new evidence on how people experience their own health condition and health and well-being is to be generated. Robust reporting is essential to the process of synthesis, and for new interpretations to be generated ( France *et al*., 2019a), therefore the eMERGe reporting guideline, aimed at increasing the transparency and completeness of conducting and reporting a meta-ethnography guided the development and preparation of this protocol ( France *et al*., 2019a).

## PHASE 1 - Selecting meta-ethnography and getting started

Phase one of a meta-ethnography involves reporting the rationale and the context for the study. To the best of our knowledge, no meta-ethnography exists to date, which has synthesised the child and young person’s experience of living with DCD. Therefore, the predominant reasons for choosing a meta-ethnographic approach in this review was that it would enable the researchers to develop a conceptual understanding of children’s subjective experiences of everyday life and living with DCD. This systematic review is registered with the International Prospective Register of Systematic Reviews (PROSPERO): registration number CRD42019129178.

## PHASE 2 - Deciding what is relevant

### Search strategy

There is no methodological agreement concerning the need to search for all possible articles to complete a ‘good’ qualitative synthesis ( Toye *et al*., 2014). However, for this review, we chose to complete an extensive systematic search strategy for ten databases, Academic Search Complete, AMED, CINAHL, ERIC, MEDLINE, PsychArticles, PsychInfo, EMBASE, SPORTDiscus, and Web of Science. The rationale being that we wanted to capture all possible qualitative studies that have examined children and young people’s perspectives of living with DCD. We did not envisage a large volume of papers that is 40 or more articles. However, it was deemed necessary to capture a wide range of studies, in order to obtain enough data representing children’s experiences, and allow robust conceptual categories to be developed ( Toye *et al*., 2014). Booth (2016) recognised the challenges associated with searching grey literature. Describing a time-consuming process with the potential for marginal follow up of the unpublished literature ( Booth, 2016). For these reasons, the authors chose not to include grey literature sources. Theses were not included as they have the potential to “swamp” data from naturally thinner studies ( Booth, 2016). However, the authors will search for published articles resulting from theses identified in the search.

To complement, the clarity and reporting of the search strategy and procedures, we used the Preferred Reporting Items for Systemic Reviews and Meta-Analysis Protocols (PRISMA-P) checklist ( Moher *et al*., 2015). A thorough search string was formulated based upon the comprehensive review of DCD literature by Smits-Engelsman *et al*. (2018), and a review paper focused on searching for qualitative research ( Booth, 2016). The keywords used were “Developmental Coordination Disorder/DCD” and “qualitative research” alongside thesaurus and Medical Subject Headings terms (MeSH). A librarian from the University of Limerick reviewed the search strategy and provided guidance. The search strategy used in MEDLINE is presented as an example ( Table 1).

**Table 1.** MEDLINE search strategy.

**S1**	Motor Skills Disorder* OR developmental coordination disorder OR clumsiness OR clumsy OR in-coordination OR dys- coordination OR minimal brain dysfunction OR minor neurological dysfunction OR motor delay disorder OR perceptual- motor impairment OR motor coordination difficulties OR motor learning difficulties OR mild motor problems OR non-verbal learning disability OR non-verbal learning disorder OR non-verbal learning dysfunction OR motor coordination problems OR sensorimotor difficulties OR sensory integrative dysfunction OR physical awkwardness OR physically awkward OR psychomotor disorders OR motor control and perception OR developmental dyspraxia OR perceptual motor dysfunction OR minimal cerebral dysfunction
**S2**	qualitative OR experience* OR perception* OR perspective* OR case stud* OR interview* OR focus group* OR mixed methods OR participant observation OR transcript* OR ethnograph* OR phenomenol* OR grounded theor* OR grounded- theor* OR purposive sample OR lived experience* OR narrative* OR life experience* OR life stor* OR action research OR observational method OR thematic analysis OR narrative analysis OR field stud* OR field-notes OR videorecording
**S3**	child OR children OR adolescent OR teen OR teenager OR youth OR young person OR young adult
**S4**	S1 AND S2 AND S3

### Study selection

The SPIDER (Sample, Phenomenon of Interest, Design, Evaluation, and Research type) search strategy tool helped to structure the criteria developed to screen studies, firstly by title and abstract and, subsequently, by full-text review ( Cooke *et al*., 2012). Table 2 outlines each aspect of SPIDER and inclusion/exclusion criteria. Included studies will describe a sample of children aged five to eighteen years with a diagnosis of DCD or probable DCD according to the Diagnostic and Statistical Manual of Mental Disorders 5 ^th^ Edition (DSM-V) criteria ( APA, 2013). Where children are described as having probable DCD, the authors of studies must outline the profile of participants so that categorisation of how each criterion of the DSM-V was fulfilled can be evaluated.

**Table 2.** Inclusion and exclusion criteria.

	Inclusion Criteria	Exclusion criteria
**Sample**	Children aged five to eighteen years with a diagnosis of DCD or probable DCD. Participants with DCD and a co-occurring specific learning difficulty or neurodevelopmental diagnosis such as ADHD will be included as co-occurrence is very common ( Blank *et al*., 2019). Participants must meet the Diagnostic and Statistical Manual of Mental Disorders 5th Edition (DSM-V) criteria for DCD. Where children and young people are described as having probable DCD, the authors must outline how each criterion of the DSM-V was fulfilled: 1. motor impairment scores below the 15 ^th^ percentile on a standardised motor test; 2. describe how the participants’ activities of daily living are affected as a result of the motor skills difficulties 3. explain the participants cognitive ability and confirm that it is within the normal intellectual ranges 4. indicate that no underlying medical condition is reported by parents, guardians, teachers or health professionals. Studies examining parental and child experiences will be included, but it must be possible to extract data on the child and young person views and experiences of living with DCD.	Children younger than five years will be excluded as a diagnosis of DCD is not confirmed below five years of age ( Blank *et al*., 2019). Studies that include a sample of children and young people with a different diagnosis will be excluded if it is not possible to extract the views and experiences of children and young people wit DCD within such studies. Studies examining the opinions and experiences of parents of children with DCD will be excluded.
**Phenomenon** **of interest**	Children and young people who describe their views, opinions and experiences of living with DCD.	
**Design**	Qualitative or mixed-methods studies reporting primary qualitative data (e.g., data collected through qualitative methods such as interviews, focus groups, or participant observation etc.)	Where the qualitative data from the child cannot be identified, such as summaries or aggregated data of parent and child experiences, these papers will be excluded.
**Evaluation**	Qualitative analysis of experiences, feelings, views, opinions, and experiences of living with DCD. All settings such as school, home, community, etc. will be included.	Studies where a method of qualitative analysis is not described.
**Research type**	Peer-reviewed journal articles and thesis. Full text available in English Published between No date limit- 2019	Systematic reviews, protocols, theoretical work, editorials, opinion pieces and dissertations, grey literature.

1. Motor impairment scores are recorded as less than the 15 ^th^ percentile on a standardised motor test.
2. Describe how the participants’ everyday activities are affected because of the motor skills difficulties.
3. Explain the participants cognitive ability and confirm that it is within the normal intellectual ranges.
4. Indicate that no underlying medical condition is reported by parents, guardians, teachers, or health professionals.

Participants with DCD and a co-occurring specific learning difficulty or neurodevelopmental diagnosis such as Attention Deficit Disorder Hyperactivity will be included as co-occurrence is common ( Blank *et al*., 2019). Furthermore, studies examining parental and children’s views will be included, but it must be possible to extract the data on the child’s views and experiences of living with DCD, as the phenomenon of interest under investigation is children and young people’s views, opinions, and experiences of everyday life and living with DCD. All studies using a qualitative design, including mixed methods studies that report extractable qualitative data from the child’s perspective, will be included. The setting of the study will not be limited. All peer-reviewed articles published in English will be included. Due to pragmatic reasons of time and the financial burden associated with translation, searches will be limited to English publications only. No date limit will be applied to the search to capture all possible citations.

Studies will be excluded, if (a) they include a sample of children with a range of neurodevelopmental diagnoses and the qualitative data for the children with DCD cannot be extracted, or (b) the data presented is aggregated (for example, a mix of parent and child data that cannot be easily identifiable). Finally, systematic reviews, study protocols, and theses will be excluded.

Once, the search strategy has been completed in each of the identified databases, the citations retrieved will be uploaded to Endnote software and the duplicate citations removed. These citations will be exported to Rayyan software, to facilitate the screening of the papers by title and abstract ( Ouzzani *et al*., 2016). Whilst, quantitative synthesis recommend a prescribed requirement for two reviewers to screen articles, qualitative synthesis do not share this requirement as a protection against bias ( Booth *et al*., 2013). Instead, reviewer resources could be employed more efficiently, to enhance the quality of analysis and interpretation ( Booth *et al*., 2013). Therefore, 10% of papers will be screened by title and abstract to check for consistency by KR. If there is any ambiguity about an article title or abstract, it will be added for full-text review. Two independent reviewers (ÁOD and KR) will use the selection criteria to conduct a full-text review for all included papers. Where any discrepancies arise at the full-text review stage, these differences will be resolved through discussion. If it is challenging to resolve differences of opinion, a third reviewer (SC) will help to facilitate a final decision. The PRISMA-P flowchart will be populated to present the results generated at each stage of the process ( Moher *et al*., 2015).

### Quality appraisal of the included studies

This meta-ethnography aims to add to the conceptual understanding of living with DCD from the child and young person’s perspective so that it can inform practice, research and policy; therefore, the studies included in this qualitative evidence synthesis must be ‘good enough’ ( Toye *et al*., 2013). Toye *et al*. (2013) present a conceptual model of quality, which centres on conceptual clarity and interpretive rigour; and the researchers advocate the need for such a model to be used when completing meta-ethnography. The two principal features are defined as 1) “Conceptual clarity (how has the author articulated a concept that facilitates theoretical insight)”, and 2) “Interpretive rigour (What is the context of interpretation? How inductive are the findings? Has the interpretation been challenged?)” ( Toye *et al*., 2013). In line with this conceptual model of quality, we have selected the Joanna Briggs Institute (JBI) Checklist for Qualitative Research ( Joanna Briggs Institute, 2017) to appraise all included papers. The JBI checklist is the most sensitive tool when examining methodological validity, given its focus on congruity including descriptive, interpretative, theoretical, external and evaluative validity ( Hannes *et al*., 2010).

All included papers will be critically appraised by two independent reviewers (ÁOD and KR) using the JBI Checklist ( Joanna Briggs Institute, 2017). The JBI tool will be used to inform judgements about the methodological quality of the articles; decisions will be categorised as ‘include’ or ‘exclude’ and comments on the decisions will be recorded. The outcomes of the critical appraisal process will be compared and any variances in decisions will be discussed to reach consensus on the appraisal. If the involvement of a third reviewer is necessary, SC will contribute to the final decision-making process. In light of the quality appraisal results, the synthesis and interpretation of the included studies will be discussed.

## PHASE 3—Reading included studies

### Data extraction and synthesis

The analytical and synthesis process in meta-ethnography commences by reading the studies, described as phase three by France *et al*. (2019a) and Noblit & Hare (1988). Reading and re-reading the studies in depth is a fundamental aspect to data extraction and continues to be an iterative process during data extraction and synthesis ( Toye *et al*., 2014). The views, perceptions, or concepts presented in the results and discussion of primary studies are considered the raw data of meta-ethnography ( Toye *et al*., 2014). These concepts and ideas are labelled as second-order constructs and are derived from the researcher’s analysis and interpretation of the research participants words used to describe their experiences of the phenomenon such as living with DCD, also known as first-order constructs or key concepts ( Toye *et al*., 2014).

Previous authors have emphasised the importance of deciding what data to extract, and process of completion ( Toye *et al*., 2014; Wong *et al*., 2018). In this review, two independent reviewers will use a Microsoft Excel sheet to collate information on the characteristics of each study, such as citation, study setting/country, sample size, participant characteristics, aims of the study, data collection and methods, and summary of findings. ÁOD will also upload a PDF of each paper to QSR International’s NVivo 12 software. The first- and second-order constructs will be extracted and interpreted; the researchers (AOD and KR) will generate codes that describe and explain the key concepts within each study. NVivo software will provide an organised database through which interpretation can be completed. The researchers ÁOD and KR will code second-order findings as they present within each paper. These interpretations and synthesis of the second-order constructs become the third-order constructs ( Noblit & Hare, 1988). No second-order constructs that are considered unrelated to the phenomena or experience of living with DCD will be included for synthesis ( Toye *et al*., 2014).

## PHASE 4 - Determining how studies are related

Phase four of meta-ethnography involves determining how studies are related ( France *et al*., 2019a). Following coding of second-order constructs, ÁOD and KR will meet regularly to discuss and compare their concepts and determine how the studies relate to each other, and the review question ( France *et al*., 2019a) and will involve Mandy Stanley (MS) an invited expert in the area of meta-ethnography at this and subsequent stages. These meetings will aim to challenge the interpretation of concepts and compare them across each study. This method of identifying the similarities and differences, across the included studies will be a prerequisite step that informs the “translation” process described as phase five by ( Noblit & Hare, 1988).

## PHASE 5 - Translating studies into one another

Phase five; the next stage will involve translating studies into each other ( Noblit & Hare, 1988). France *et al*. (2019a) suggest that translation can be performed in different ways. In this review, the authors will follow a method described by Toye *et al*. (2014). Toye & colleagues (2014) suggest that constructs should be constantly compared until similarities and differences between concepts can be organised into conceptual categories to represent the third-order constructs. Given that the sample of children and young people included in this study is 5 to 18 years, the primary studies may report a variety of experiences depending upon the age of the included sample. It will be essential to preserve the context and meaning of the identified concepts during the translation concerning any subgroups such as age, as recommended by Campbell *et al*. (2003). For this reason, the method of constant comparison across studies was deemed more appropriate rather than translating studies in chronological order ( Toye *et al*., 2014).

Once preliminary conceptual categories are created, ÁOD will present the findings to the broader research team, including SC and MS. Through discussions, these third-order constructs will be further developed and refined.

## PHASE 6 & 7 Synthesizing translations and Expressing the synthesis

The final stages, phase six and seven, will involve the research team synthesising the conceptual categories into a line of argument, which provides greater conceptual understanding to the phenomena of interest as a whole; that is children and young people with DCD perspectives and experiences of everyday life and living with DCD. The conceptual categories and line of argument synthesis will be presented narratively; tables and figures will be created to support the narrative account. The findings of this meta-ethnography endeavour to inform future research, policy and practice. Therefore, dissemination will involve the publication of the results in a peer-reviewed journal. An infographic designed policy brief will be published, to capitalise on knowledge translation and target a broader audience of policymakers, service providers, and clinicians. The policy brief will be distributed to advocacy groups who work on behalf of children and young people with DCD. Knowledge translation is challenging; in the context of childhood disability it is imperative that findings are easily accessible and usable ( Novak & Honan, 2019). Given the national and international focus upon promoting the voice of the child, the findings of this study must be presented in an easily accessible format for all possible stakeholders ( Ombudsman for Children's Office, 2019).

## Discussion

### Limitations and strengths

To the best our knowledge, we believe this is the first systematic review to integrate and synthesise the findings of qualitative studies on the views and experiences of children and young people living with DCD. The findings of this review will be relevant for researchers, practitioners, and policymakers working with children and young people with DCD. Given that there is a paucity of evidence regarding effective interventions to improve participation outcomes for children with DCD ( Novak & Honan, 2019; O’Dea *et al*., 2019), the results of this review will add to the empirical evidence when designing a complex intervention for children with DCD to improve participation in everyday life. Thus, adding to research knowledge and reducing research waste by synthesising and conceptualising available evidence that can be used in the development of a complex intervention ( Bleijenberg *et al*., 2018).

Addressing rigour is an essential aspect for the qualitative researcher. It is necessary to recognise that ÁOD is a PhD scholar and an Occupational Therapist who has worked clinically with children and young people with DCD. The other members of the research team have extensive research experience in a range of methodologies. It is envisaged that the meetings to discuss the analysis and interpretation of results will challenge any possible pre-existing assumptions that may influence results.

## Data availability

### Underlying data

No data are associated with this article.

### Reporting guidelines

Figshare: PRISMA-P checklist for "Children and young people's experiences of living with developmental coordination disorder/ dyspraxia: study protocol for a qualitative evidence synthesis”. https://doi.org/10.6084/m9.figshare.10002788.v2 ( O’Dea *et al*., 2019).
