# Supplementary material for: Children and young people's experiences of living with developmental coordination disorder/dyspraxia: study protocol for a qualitative evidence synthesis
Source: HRB Open Res. 2021 Jan 18;2:28. Originally published 2019 Oct 28. [Version 4] doi: 10.12688/hrbopenres.12958.4 (PMC7017181; doi:10.12688/hrbopenres.12958.4)
Supplement: Peer review history

# Peer review: Reviewer response for version 4

**DOI:** 10.21956/hrbopenres.14357.r28664

**Published:** 2021-01-25

**Authors:** Motohide Miyahara, Tessa Pocock

**Affiliations:** 1 Community Heath, School of Nursing, University of Auckland, Auckland, New Zealand; 2 Department of Clinical Psychological Science, School of Medicine, Hirosaki University, Hirosaki, Japan

We are happy with the final revision and grateful for the authors who have sincerity taken our comments on board to the end. Congratulations!

Is the study design appropriate for the research question?

Yes

Is the rationale for, and objectives of, the study clearly described?

Yes

Are sufficient details of the methods provided to allow replication by others?

Yes

Are the datasets clearly presented in a useable and accessible format?

Not applicable

Reviewer Expertise:

Qualitative meta-synthesis.

We confirm that we have read this submission and believe that we have an appropriate level of expertise to confirm that it is of an acceptable scientific standard.

# Peer review: Reviewer response for version 3

**DOI:** 10.21956/hrbopenres.14266.r28154

**Published:** 2020-11-04

**Authors:** Motohide Miyahara, Tessa Pocock

**Affiliations:** 1 Department of Clinical Psychological Science, School of Medicine, Hirosaki University, Hirosaki, Japan; 2 Community Heath, School of Nursing, University of Auckland, Auckland, New Zealand

The authors took the reviewers’ comments seriously and addressed most of the issues raised.

 The third paragraph in the introduction improved its accuracy, but it is still confusing as it is. If the authors want to present competing views, they need to introduce the existence of the different views in the first topic sentence. It is also helpful for the reader to explain what creates the two completely opposing views on the exiting evidence on the intervention effect. The key issue may be the quality of evidence (Miyahara, Lagisz, Nakagawa, & Henderson, 2020) ^1^.

 The authors use the term evidence in the third and fifth paragraphs, but the meanings are quite different between the paragraphs. The authors are encouraged to use a different term in the fifth paragraph.

 The authors adequately justified the reasons for focusing on children’s view in terms of intervention development.

 The sixth paragraph justify the study of children’s views well, but the use of meta ethnography is not explained in the seventh paragraph. In the second sentence in the method section, the authors explain that they chose meta-ethnography because it is interpretative rather than aggregative. The reader may wonder, if they understand the difference between interpretive and aggregative meta analysis, why it is important to choose the interpretive method of synthesis rather than aggregative methods. Please explain fully.

 Please also carefully check the formatting of in-text citations and have a very good look at grammar, including punctuations (e.g., colons and semicolons).

Is the study design appropriate for the research question?

Yes

Is the rationale for, and objectives of, the study clearly described?

Yes

Are sufficient details of the methods provided to allow replication by others?

Yes

Are the datasets clearly presented in a useable and accessible format?

Not applicable

Reviewer Expertise:

Qualitative meta-synthesis.

We confirm that we have read this submission and believe that we have an appropriate level of expertise to confirm that it is of an acceptable scientific standard, however we have significant reservations, as outlined above.

## References

[1] : Intervention for children with developmental coordination disorder: How robust is our recent evidence?. *Child Care Health Dev*.2020;46(4) : 10.1111/cch.12763 397-406 10.1111/cch.12763 32167189

# Peer review: Reviewer response for version 2

**DOI:** 10.21956/hrbopenres.14222.r27776

**Published:** 2020-08-13

**Authors:** Motohide Miyahara, Tessa Pocock

**Affiliations:** 1 Department of Clinical Psychological Science, School of Medicine, Hirosaki University, Hirosaki, Japan; 2 Community Heath, School of Nursing, University of Auckland, Auckland, New Zealand

The authors did amend the introduction, accurately reflecting the conclusion by Miyahara et al. (2017). However, this is a topic sentence of the paragraph and the rest of the paragraph does not follow well without adjusting the entire paragraph. In fact, the whole introduction is rather fragmented. As the other reviewer suggests, there is a room for improvement in the introduction in terms of justifying the study theoretically, practically, and methodologically. The current introduction still appears sketchy, missing the explanations for why children’s perspective is important and why a qualitative synthesis needs to be conducted by meta ethnography. The minor points have been addressed in a satisfactory manner.

Is the study design appropriate for the research question?

Yes

Is the rationale for, and objectives of, the study clearly described?

Yes

Are sufficient details of the methods provided to allow replication by others?

Yes

Are the datasets clearly presented in a useable and accessible format?

Not applicable

Reviewer Expertise:

Qualitative meta-synthesis.

We confirm that we have read this submission and believe that we have an appropriate level of expertise to confirm that it is of an acceptable scientific standard, however we have significant reservations, as outlined above.

# Peer review: Reviewer response for version 1

**DOI:** 10.21956/hrbopenres.14040.r27052

**Published:** 2020-02-03

**Authors:** Rob Brooks

**Affiliations:** 1 School of Clinical and Applied Sciences, Leeds Beckett University, Leeds, UK

Thank you for the opportunity to review this paper. The authors begin to provide a justification for the need for this review study and outline a comprehensive approach to their methodology. There are a number of strengths to this study and some areas that could benefit from further consideration. 

**Abstract**

 The abstract provides a brief overview of the study. This includes some detail of background, methodology and outcomes. 

*Abstract specific feedback:*

- In the abstract and in the introduction the authors refer to ‘In the past…’ it would be helpful to know if this a reference to past research? 
- There is an interchangeable use of the term’s children and children and young people – it would be good to be consistent.
- The abstract mentions experience of relationships but this idea is not really brought through in to the main introduction. 
- There is an overuse of semi-colons, shorter sentences may address this.

**Introduction**

 Overall, the authors draw on a range of literature and offer a gap in knowledge for their study. The presentation of DCD and its consequences are clearly presented. There are a number of terms used related to the ICF, including activities, body functions and participation. Use of the ICF in the paper may help situate the discussion more clearly. There is perhaps an over presentation/discussion of interventions for DCD, which is not the focus of this study. The paper describes a ‘pressing need’ and that is ‘timely’ for this study but offers limited justification for this.

*Introduction specific feedback:*

- The authors list specific everyday activities - dressing, feeding, writing, but then also include broader taxonomies – self-care and leisure. Consider greater constancy. 
- Is DCD prevalence universal, are there any international studies that could be drawn on?
- The authors refer to recent systematic reviews and meta-analyses – (plural) but you only reference one of each. Consider revision.
- There are a number of references missing from the reference list, for example Wilson *et al*., 2013 and Hana and Rodgers, 2002. 

**Methods and related sections**

 This is section is an area of strength for the paper. The authors have shown clarity and been methodical. The research design is appropriate to the study objective. Some of the information in this section repeats information in Table 2 which may not be needed. There is a clear strategy for study selection, quality appraisal, and data extraction. 

*Methods specific feedback:*

- The authors describe how meta-ethnography has become popular – are there any relevant studies that demonstrate this?
- You are using a range of databases but not appear to be any relating to education (such as ERIC), where experiences of school could be published.
- Consider the addition of the term juvenile to the search criteria.
- The inclusion of studies where the participants with probable DCD needs justification. 
- Your review of title and abstract by only one reviewer should be reconsidered. 
- Please check the reference for the JBI Qualitative methodology checklist.

Is the study design appropriate for the research question?

Yes

Is the rationale for, and objectives of, the study clearly described?

Partly

Are sufficient details of the methods provided to allow replication by others?

Yes

Are the datasets clearly presented in a useable and accessible format?

Not applicable

Reviewer Expertise:

Occupational therapy, paediatrics, child and adolescent mental health, qualitative research.

I confirm that I have read this submission and believe that I have an appropriate level of expertise to confirm that it is of an acceptable scientific standard.

# Response

**Published:** 2020-07-23

**Authors:** Aine O'Dea

**Affiliations:** Ms, Ireland

**Reviewer 2- Comment 1**

In the abstract and the introduction the authors refer to ‘In the past…’ it would be helpful to know if this a reference to past research?

**Response:** The authors have clarified and rephrased this sentence in the abstract.

**Reviewer 2- Comment 2**

There is an interchangeable use of the term’s children and children and young people – it would be good to be consistent.

**Response: **Consistency of terms has been addressed throughout.

**Reviewer 2- Comment 3**

The abstract mentions experience of relationships, but this idea is not really brought through into the main introduction.

**Response: **We have amended the abstract to remove reference to ‘relationships’ and re-orientate that sentence towards children’s experiences in general.  

**Reviewer 2- Comment 4**

There is an overuse of semi-colons, shorter sentences may address this.

**Response: **This has been addressed.

**Reviewer 2- Comment 5**

The authors list specific everyday activities - dressing, feeding, writing, but then also include broader taxonomies – self-care and leisure. Consider greater constancy.

**Response:** The authors have rephrased that sentence in the introduction to distinguish between specific activities and broader categories of activities

**Reviewer 2- Comment 6**

Is DCD prevalence universal; are there any international studies that could be drawn on?

**Response:** Prevalence is discussed in a little further detail to acknowledge variation in international prevalence rates.

**Reviewer 2- Comment 7**

The authors refer to recent systematic reviews and meta-analyses – (plural), but you only reference one of each. Consider revision.

**Response:** The authors have corrected the phrasing.

**Reviewer 2- Comment 8**

There are a number of references missing from the reference list, for example, Wilson *et al*., 2013 and Hanna and Rodgers, 2002.

**Response:** The missing references have been added

**Reviewer 2- Comment 9**

The authors describe how meta-ethnography has become popular – are there any relevant studies that demonstrate this?

**Response:** The authors have added the reference to highlight the popular use of meta-ethnography in health services research.

**Reviewer 2- Comment 10**

You are using a range of databases but not appear to be any relating to education (such as ERIC), where experiences of school could be published.

**Response**

We recognise that searching ERIC could yield further relevant studies. As such, we have included this database.

**Reviewer 2- Comment 11**

Consider the addition of the term juvenile to the search criteria.

**Response:** We recognise that adding the term juvenile may have been helpful. We will acknowledge the omission of this search term as limitations when we prepare the synthesis for publications.

**Reviewer 2 – Comment 12**

The inclusion of studies where the participants with probable DCD needs justification.

**Response:** We have clarified that where children are described as having probable DCD, the authors of studies must outline the profile of participants. So that categorisation of how each criterion of the DSM-V was fulfilled can be evaluated.

**Reviewer 2- Comment 13**

Your review of title and abstract by only one reviewer should be reconsidered.

**Response:** While quantitative synthesis recommends a prescribed requirement for two reviewers to screen articles, qualitative synthesis does not share this requirement as protection against bias (Booth *et al.*, 2013). Instead, reviewer resources could be employed more efficiently, to enhance the quality of analysis and interpretation (Booth *et al.*, 2013). Therefore, 10% of papers will be screened by title and abstract to check for consistency by KR, now stated in the ‘study selection’ section.

**Reviewer 2- Comment 14**

Please check the reference for the JBI Qualitative methodology checklist.

**Response:** This has been addressed

# Peer review: Reviewer response for version 1

**DOI:** 10.21956/hrbopenres.14040.r26940

**Published:** 2019-12-03

**Authors:** Motohide Miyahara, Tessa Pocock

**Affiliations:** 1 Department of Clinical Psychological Science, School of Medicine, Hirosaki University, Hirosaki, Japan; 2 Community Heath, School of Nursing, University of Auckland, Auckland, New Zealand

It is an honour and privilege to be asked to review this protocol. I am invited probably because I co-authored one of the first qualitative synthesis studies concerning young people with disability with my former student, Tessa Pocock who served as the first author for the study (Pocock & Miyahara, 2017 ^1^) which derived from her Honour’s dissertation. Currently a PhD student, Tessa expressed her interest to learn how to peer-review a paper, so I invited her to be a co-reviewer. When she decided to perform qualitative meta-synthesis, I assigned her to read a book entitled, “Meta-Study of Qualitative Health Research” by Paterson, Thorne, Canam, and Jillings (2001) ^2^. This book contained the information about meta-ethnography which is planned to be used in O’Dea, Coote, and Robinson’s proposed study.

 The study protocol starts with the brief account of children with developmental coordination disorder (DCD), followed by the impact of DCD on children’s life participation and health. To counter the negative impact of DCD, the contemporary health service delivery model targets children’s perspectives on goal-setting in participating in desirable life situations. This approach can benefit from qualitative studies tapping into the experiences of children with DCD. However, the number of such studies are limited and hardly synthesized. Therefore, the proposed study is worth conducting not only to fill the research gap, but also to inform the health service providers to develop and implement appropriate service. After searching, selecting, and extracting data from target studies, the meta-ethnographic approach will be applied to synthesize the data, following the process specified by Noblit and Hare (1988) ^3^.

 The protocol is written clearly with a well-justified rationale and purpose. While the choice of meta-ethnography is appropriate for the purpose, the reader who is unfamiliar with the particular approach would benefit from further explanation, starting with a clarification of why this particular approach was selected, whether meta-ethnography is applied to ethnographic studies or other types of qualitative studies, and some basic tenets of meta-ethnography. The planned process is consistent with the steps illustrated by Noblit and Hare (1988) ^3^ and the protocol authors should be able to carry out the protocol as they plan.

 Taking advantage of this opportunity, we would like to point out one minor yet significant discrepancy. At the beginning of the third paragraph of Introduction, the protocol authors state that “task-oriented interventions are effective” citing Miyahara, Hillier, Pridham, and Nakagawa (2017) ^4^ and Smits-Engelsman *et al*. (2018) ^5^. Although the latter review concludes as such, the former review maintains that there is no strong evidence that supports the efficacy of task-oriented approach, which is more consistent with the second sentence than the review by Smits-Engelsman *et al*. (2018) ^5^. The protocol authors are encouraged to amend these two sentences in the final report which we very much look forward to reading.

 In addition, we suggest slight modification and/or clarification be made to the following points:

 Introduction

- Third paragraph: Provide reference details for “Hanna & Rodgers, 2002” citation.

Methods

- To be consistent with the abstract, clarify how the eMERGe reporting guidelines will be used in the rest of the review process.

Search strategy

- First sentence, second line: comma is not necessary after “search for”.
- Alongside the nine listed databases, please clarify whether grey literature is also being searched for. If not, please justify/state why.
- Please clarify whether a librarian was consulted during the development of the search strategy.

Study selection

- Fourth paragraph: What is the rationale for having two independent reviewers screen only the full text? Can an additional reviewer assist with all or a set number of the title and abstract screening, too?

Table 2

- Inclusion criteria, Sample, point 3: “cognitively ability” should be ‘cognitive ability’. This change should be made in the text under the table as well.
- Exclusion criteria, Research type: should grey literature be listed here? Do the protocol authors consider opinion pieces and dissertations as grey literature?

Quality appraisal of the included studies

- Please clarify *what* should be congruent in the sentence: “The JBI checklist is the most sensitive tool when examining methodological validity, given its focus on **congruity**.”

Data extraction and synthesis

- First paragraph, first sentence: Remove brackets from the reference to France et al., 2019; Noblit & Hare, 1988.
- Fourth paragraph, third sentence: Remove brackets from the reference to Toye et al., 2014.
- Fourth paragraph, sixth sentence: Remove brackets from the reference to Campbell et al., 2003.
- Fifth paragraph, first sentence: Remove comma after “Once”.
- Fifth paragraph, first sentence: Would it also be helpful to have the invited expert involved earlier in the meta-ethnography process, rather than just looking at the preliminary findings (if possible)?
- Sixth paragraph, third sentence: Remove comma from after “meta-ethnography”.
- Sixth paragraph, eighth sentence: “Nation” should be ‘national’.

Is the study design appropriate for the research question?

Yes

Is the rationale for, and objectives of, the study clearly described?

Yes

Are sufficient details of the methods provided to allow replication by others?

Yes

Are the datasets clearly presented in a useable and accessible format?

Not applicable

Reviewer Expertise:

Qualitative meta-synthesis.

We confirm that we have read this submission and believe that we have an appropriate level of expertise to confirm that it is of an acceptable scientific standard.

## References

[1] : Inclusion of students with disability in physical education: a qualitative meta-analysis. *International Journal of Inclusive Education*.2018;22(7) : 10.1080/13603116.2017.1412508 751-766 10.1080/13603116.2017.1412508

[2] : Meta-Study of Qualitative Health Research. *Sage Publishing*.2001;

[3] Meta-ethnography: Synthesizing qualitative studies. *Sage Publishing*.1988;

[4] : Task-oriented interventions for children with developmental co-ordination disorder. *Cochrane Database of Systematic Reviews*.2017; 10.1002/14651858.CD010914.pub2 10.1002/14651858.CD010914.pub2 PMC648334428758189

[5] : Evaluating the evidence for motor-based interventions in developmental coordination disorder: A systematic review and meta-analysis. *Res Dev Disabil*.2018;74: 10.1016/j.ridd.2018.01.002 72-102 10.1016/j.ridd.2018.01.002 29413431

# Response

**Published:** 2020-07-23

**Authors:** Aine O'Dea

**Affiliations:** Ms, Ireland

**Reviewer 1 - Comment 1.** “While the choice of meta-ethnography is appropriate for the purpose, the reader who is unfamiliar with the particular approach would benefit from further explanation, starting with a clarification of why this particular approach was selected, whether meta-ethnography is applied to ethnographic studies or other types of qualitative studies, and some basic tenets of meta-ethnography”.

**Response**: In the methods section, an enhanced description of the approach is presented regarding an introductory article describing the approach (Cahill et al., 2018).

**Reviewer 1- Comment 2**. “We would like to point out one minor yet significant discrepancy. At the beginning of the third paragraph of Introduction, the protocol authors state that “task-oriented interventions are effective” citing Miyahara, Hillier, Pridham, and Nakagawa (2017) and Smits-Engelsman et al. (2018). Although the latter review concludes as such, the former review maintains that there is no strong evidence that supports the efficacy of task-oriented approach, which is more consistent with the second sentence than the review by Smits-Engelsman et al. (2018). The protocol authors are encouraged to amend these two sentences in the final report, which we very much look forward to reading.

**Response: **We have amended this section to acknowledge that the evidence regarding the efficacy of interventions to treat DCD is not clear (Miyahara *et al.*, 2017) and clarified that the  Cochrane  review by Miyahara and colleagues found that no strong evidence exists that supports the efficacy of task-oriented interventions for children and young people with DCD

**Reviewer 1- Comment 3. **To be consistent with the abstract, clarify how the eMERGe reporting guidelines will be used in the rest of the review process.

**Response: **The authors have amended the layout of the protocol so that each phase of the seven phases are attended to under the appropriate heading. Thus reflecting the layout of the eMERGe reporting guidelines and how the review will be conducted.

**Reviewer 1- Comment 4 “**Alongside the nine listed databases, please clarify whether grey literature is also being searched for. If not, please justify/state why”

**Response**: Booth (2016) recognised the challenges associated with searching grey literature. Describing a time-consuming process with the potential for marginal follow up of the unpublished literature (Booth, 2016). For these reasons, the authors chose not to include grey literature sources. This is now explicitly stated in the search strategy section.

**Reviewer 1- Comment 5 “**Please clarify whether a librarian was consulted during the development of the search strategy”.

**Response:** A librarian from the University of Limerick reviewed the search strategy and provided guidance. This is now explicitly stated in the search strategy section.

**Reviewer 1- Comment 6 **What is the rationale for having two independent reviewers screen only the full text? Can an additional reviewer assist with all or a set number of the title and abstract screening, too?

**Response:** While quantitative synthesis recommends a prescribed requirement for two reviewers to screen articles, qualitative synthesis does not share this requirement as protection against bias (Booth *et al.*, 2013). Instead, reviewer resources could be employed more efficiently, to enhance the quality of analysis and interpretation (Booth *et al.*, 2013). 10% of papers will be screened by title and abstract to check for consistency by KR, now detailed in the ‘study selection’ section.

**Reviewer 1- Comment 7 **Exclusion criteria, Research type: should grey literature be listed here? Do the protocol authors consider opinion pieces and dissertations as grey literature?

**Response: **For the reasons stated above, we chose not to include grey literature sources. Furthermore, theses were not included as they have the potential to “swamp” data from naturally thinner studies (Booth, 2016). However, the authors will search for published articles resulting from theses identified in the search. This is now stated in the ‘study selection’ section.

**Reviewer 1- Comment 8**. Please clarify what should be congruent in the sentence: “The JBI checklist is the most sensitive tool when examining methodological validity, given its focus on congruity.”

**Response:** We have clarified this by expanding the sentence: “The JBI checklist is the most sensitive tool when examining methodological validity, given its focus on congruity including, descriptive, interpretative, theoretical, external and evaluative validity ( Hannes *et al*., 2010).”

**Reviewer 1- Comment 9 **Would it also be helpful to have the invited expert involved earlier in the meta-ethnography process, rather than just looking at the preliminary findings (if possible)?

**Response:** We have amended the plan and will now involve the invited expert for the early stages of analysis and interpretation. This is now explicitly stated in ‘Phase 4’ (page 11).
